# Iron deposition is independent of cellular inflammation in a cerebral model of multiple sclerosis

**DOI:** 10.1186/1471-2202-12-59

**Published:** 2011-06-23

**Authors:** Rachel Williams, Aaron M Rohr, Wen-Tung Wang, In-Young Choi, Phil Lee, Nancy EJ Berman, Sharon G Lynch, Steven M LeVine

**Affiliations:** 1 Department of Molecular & Integrative Physiology, University of Kansas Medical Center, Kansas City, KS 66160; 2 Hoglund Brain Imaging Center, University of Kansas Medical Center, Kansas City, KS 66160; 3 Department of Anatomy & Cell Biology, University of Kansas Medical Center, Kansas City, KS 66160; 4 Department of Neurology, University of Kansas Medical Center, Kansas City, KS 66160

## Abstract

**Background:**

Perivenular inflammation is a common early pathological feature in multiple sclerosis (MS). A recent hypothesis stated that CNS inflammation is induced by perivenular iron deposits that occur in response to altered blood flow in MS subjects. In order to evaluate this hypothesis, an animal model was developed, called cerebral experimental autoimmune encephalomyelitis (cEAE), which presents with CNS perivascular iron deposits. This model was used to investigate the relationship of iron deposition to inflammation.

**Methods:**

In order to generate cEAE, mice were given an encephalitogen injection followed by a stereotactic intracerebral injection of TNF-α and IFN-γ. Control animals received encephalitogen followed by an intracerebral injection of saline, or no encephalitogen plus an intracerebral injection of saline or cytokines. Laser Doppler was used to measure cerebral blood flow. MRI and iron histochemistry were used to localize iron deposits. Additional histological procedures were used to localize inflammatory cell infiltrates, microgliosis and astrogliosis.

**Results:**

Doppler analysis revealed that cEAE mice had a reduction in cerebral blood flow compared to controls. MRI revealed T2 hypointense areas in cEAE animals that spatially correlated with iron deposition around vessels and at some sites of inflammation as detected by iron histochemistry. Vessels with associated iron deposits were distributed across both hemispheres. Mice with cEAE had more iron-labeled vessels compared to controls, but these vessels were not commonly associated with inflammatory cell infiltrates. Some iron-laden vessels had associated microgliosis that was above the background microglial response, and iron deposits were observed within reactive microglia. Vessels with associated astrogliosis were more commonly observed without colocalization of iron deposits.

**Conclusion:**

The findings indicate that iron deposition around vessels can occur independently of inflammation providing evidence against the hypothesis that iron deposits account for inflammatory cell infiltrates observed in MS.

## Background

Recent studies suggest a possible link between iron deposition around vessels, poor venular blood flow and perivascular inflammation in the CNS of multiple sclerosis (MS) subjects [1-4]. Perivascular iron deposits have been observed both histologically [5] and by MRI susceptibility weighted imaging (SWI) [6,7]. One proposed explanation for these iron deposits is the extravasation of red blood cells (RBCs) across the BBB and their subsequent phagocytosis by macrophages [5,7]. In addition, altered blood flow has been observed both intracranially [1,2,4] and extracranially [3] in MS subjects. Extracranial vessel stenosis has been hypothesized to explain the disturbed blood flow, and the altered blood flow has been hypothesized to induce the extravasation of RBCs resulting in iron deposits that trigger inflammatory changes in the CNS [3,4,8-11]. However, alternative explanations could account for these observations. For example, altered blood flow could result from vessel congestion or occlusion due to inflammatory cells, fibrin deposits, or other factors within the brain [5,12-14], and iron deposits could develop as a consequence of inflammatory reactions rather than inducing these pathological events.

Inflammatory cell infiltrates located around CNS veins are a recurring pathological characteristic observed in MS, particularly relapsing remitting MS [12,15,16]. Despite these observations it is unknown whether iron deposition, reduced blood flow, and perivascular inflammation are interrelated. A useful way to address the interrelationship is through the study of an animal model of MS that enables systematic analyses at different stages of disease development. While there are many animal models of MS [17], very few consistently develop lesions in the cerebrum, which is the principal site where iron deposits have been described in MS [5,7-9,18-20]. One objective of the present study was to develop an animal model that generates cerebral pathology, in particular, iron deposits and vessel changes similar to those present in MS. A second objective was to utilize this model to address the interrelationship of iron deposition, perivascular inflammation and reduced blood flow.

## Methods

### Induction of cEAE

All studies involving the use of animals were approved by the Institutional Animal Care and Use Committee of the University of Kansas Medical Center. EAE was induced in 5-6 week old female SJL mice (Jackson Laboratory, Bar Harbor, ME) as previously described [21] with the exception of using a half dose of encephalitogen. The encephalitogen peptide, proteolipid protein (PLP) amino acids 139-151, was suspended in saline and emulsified in an equal volume of Freund's incomplete adjuvant containing *M. tuberculosis *H_37_RA. Briefly, female SJL mice were anesthetized with avertin and given a total dose of 75 μg PLP peptide with 150 μg *M. tuberculosis *that was divided into 3 s.c. injections on the dorsum. A tail vein injection of pertussis toxin (0.1 μg in 50 μl) was given on 0, 3, and 7 days post encephalitogen injection. Mice received food and water *ad libitum*.

At 8-11 days post encephalitogen injection, mice were anesthetized with avertin, prepped for surgery, given a craniotomy, and administered an intracerebral injection using a pulled glass pipette. The injection site was x = 2.0, y = -0.46, z = -1.8 mm relative to Bregma. The injection was given over 4 min using a Nanomite Injector Syringe Pump (Harvard Apparatus, Holliston, MA). Similar to a localized lesion EAE rat model [22], the injection consisted of TNF-α (250 ng) and IFN-γ (15 ng) (R&D Systems) in 2 μl. The control groups were: 1) no encephalitogen injection but given pertussis toxin injections + intracerebral injection of saline (NonEAE, saline); 2) no encephalitogen injection but given pertussis toxin injections + intracerebral injection of cytokines (NonEAE, cytokine); and 3) encephalitogen injection with pertussis toxin injections + intracerebral injection of saline (EAE, saline).

### MRI

Five cEAE mice were selected for MRI at either 4 or 21-23 days following the intracerebral injection. The MR imaging was performed on a 9.4 Tesla MR system, which had a Varian INOVA console (Varian Inc., Palo Alto, CA), and gradient coils (40 G/cm, 250 μs) of 12 cm in diameter (Magnex Scientific, Abingdon, UK). Anesthesia was induced with 4% isoflurane and maintained at 1%-2%. A quadrature RF surface coil was placed on top of the animal head to acquire T2-weighted spin-echo images with the following imaging parameters: TE/TR = 30/1500 ms, matrix size = 192 × 192, field of view = 12.8 × 12.8 mm, slice thickness = 0.2 mm, and number of averages = 6. The corresponding nominal image resolution was 67 × 67 × 200 μm.

### Iron histochemistry

At 4 or 21-23 days after the stereotactic injection, mice were anesthetized with isoflurane and perfused with saline followed by 10% buffered formalin (Fisher Scientific, Waltham, MA). These time points were selected since they represented periods of primary (Day 4) and secondary (Day 21-23) waves of inflammation and clinical signs. Brains were post-fixed overnight at 4°C and transferred to a cryoprotectant solution, 25% glycerol and 2.5% DMSO in PBS. Brains were embedded in 10% gelatin in cryoprotectant solution and cross linked with 10% buffered neutral formalin in cryoprotectant solution for 48 h. Coronal sections, 50 μm thick, were prepared with a microtome, stored in 50% ethylene glycol and 1% (w/v) polyvinyl pyrrolidone in PBS at -20°C. Sections were stained by iron histochemistry as described previously [23]. Briefly, sections were rinsed with PBS and incubated for 30 min at room temperature with a 1:2:1 ratio of 4% potassium ferrocyanide, 2% Triton X-100, and 0.5 N HCl respectively. The sections were then washed in PBS and incubated for 12-15 min in 0.2 mg DAB per ml of 0.01 M Tris-HCl, pH 7.4, with 0.06% H_2_O_2_. Sections were rinsed in PBS, immersed in a gelatin solution and mounted on a slide before being dehydrated and cover slipped using Permount (Fisher Scientific).

### Vessel measurements

All descending cortical vessels, perpendicular to the pial surface, were counted in a blinded manner, and vessel diameters were measured using Neurolucida (MBF Bioscience, Williston, VT). The ratio of iron stained to unstained vessels and the percent of iron stained vessels with a diameter > 40 μm were determined by averaging data collected from three sections per animal.

### Iron histochemistry and GFAP immunohistochemistry

Following iron histochemical staining, sections were incubated in blocking solution [98% Superblock (Pierce, Rockford, IL), 2% normal goat serum (Vector Laboratories, Inc., Burlingame, CA)] for 1 h, PBS 3 × 5 min, 1:5,000 rabbit anti-GFAP (Cat. No. Z0334; Dako North America, Inc., Carpinteria, CA) in 10% blocking solution in PBS for 1 h, PBS 3 × 5 min, 1:500 biotinylated goat anti-rabbit IgG (Vector) in 10% blocking solution for 1 h, PBS 3 × 5 min, Vectastain Elite ABC for 30 min, PBS 3 × 5 min, SG Peroxidase Substrate Kit (Vector) for 10 min, H_2_O, mounted on slides with gelatin, stained with hematoxylin, and dehydrated through xylenes.

For each animal, two sections between Bregma -0.70 and -2.00 were analyzed by an evaluator blinded to the animal's group. The somatosensory 1 barrel field/secondary somatosensory cortex on both hemispheres were evaluated for numbers of descending cortical vessels and whether or not they had gliosis, associated inflammatory cells and/or iron staining. Vessel-associated gliosis was defined as ≥2 astrocyte processes, abutting or adjacent to a vessel with the processes within 50 μm of each other, and the processes had at least one of the following characteristics: 1) an image analysis density reading of ≥150 (ImageJ, NIH), or 2) a diameter of at least 0.9 μm. A positive inflammatory response was defined as ≥4 inflammatory cells associated with a vessel while iron deposition was defined as brown staining associated with a vessel.

### Iron histochemistry and anti-Iba-1 immunohistochemistry

Following iron histochemical staining, sections were incubated in blocking solution for 1 h, PBS 3 × 5 min, 1:2,000 rabbit anti-ionized calcium binding adaptor molecule (Iba-1) (Cat. No. 019-19741; Wako Pure Chemical Industries, Ltd., Chuo-ku, Osaka, Japan) in 10% blocking solution in PBS for 1 h. Subsequent steps were equivalent to those described for GFAP staining except sections were not counterstained with hematoxylin.

For each animal, one section between Bregma -1.30 and -2.00 was evaluated. An evaluator, blinded to the animal grouping, took a picture of the somatosensory 1 barrel field cortex area with maximal Iba-1 staining from each hemisphere using a 20 × objective. The hemisphere with the greater staining was identified by visual inspection, and the rank order of staining density for all animals was determined independently from two additional evaluators blinded to the animals' grouping.

### Cerebral Surface Blood Flow

Animals were anesthetized with isoflurane, the skull was exposed, and mice were placed under a moorLDI Laser Doppler scanner (Moore Instruments, Axminster, UK). The distance of the laser to the skull surface and the surface area scanned, 44 mm^2 ^covering 2,772 pixels, were kept constant between animals. The scans were analyzed by the ImageJ histogram function, wherein each pixel was assigned a color that represented relative flow. The colors were categorized into 16 different bins which were separated by equal intervals of velocities, except for the first and last bins, which included the maximum and minimum limits of detection, respectively. Total blood flow in the cranial surface area per animal was determined by multiplying pixel numbers by their rank and summing the results from all bins.

### Statistics

Cerebral surface blood flow and astrocyte gliosis data were evaluated using the Student's T-test while counts of unstained and stained vessels following iron histochemistry, diameters of iron stained vessels, and rank ordering of microgliosis staining were evaluated using the Wilcoxon test.

## Results

### MRI T2 hypointense regions

**Figure 1:**
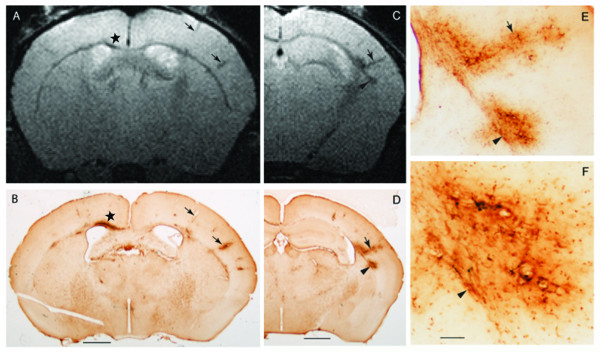
**Colocalization of T2 hypointense areas and iron deposits**. **(A) **MRI reveals an abnormal T2 hypointensity in the ipsilateral subcortical white matter (WM) (beneath the star) relative to the injection. Additional hypointense structures perpendicular to the pial surface (arrows) were present in the contralateral hemisphere. **(B) **Iron histochemical staining reveals iron deposits that colocalize with T2 hypointensities observed in A. The region beneath the star indicates an area of subcortical WM with labeling in inflammatory cells, around vessels, within microglia and generally throughout the region. The arrows indicate labeling around descending vessels that extend towards the subcortical WM. **(C) **A more posterior slice from A. T2 hypointense areas are seen in the contralateral subcortical WM (e.g., arrowhead) and in a perpendicular structure that follows the pattern of a descending cortical vessel (arrow). **(D) **Iron histochemical staining reveals a colocalization of iron deposits and T2 hypointense areas observed in C. **(E, F) **Higher magnifications of stained areas observed in D (note, arrows and arrowheads label equivalent structures in C-F). Staining around vessels occurred in endothelial cells, macrophages/microglia and/or neuropil. Bar = 1 mm for A-D. Bar in F = ~175 μm for E; ~50 μm for F.

In mice with cEAE, MRI revealed abnormal T2 hypointense regions at the injection site and in areas remote from the injection site, e.g., in the opposite cerebral hemisphere (Figure 1). The abnormal T2 hypointense sites were present in the subcortical white matter and distributed throughout the cerebral cortex. Hypointense areas in the cortex often revealed a column perpendicular to the cortical surface with a pattern following that for descending cortical vessels.

### Distribution of abnormal iron deposits

**Figure 2:**
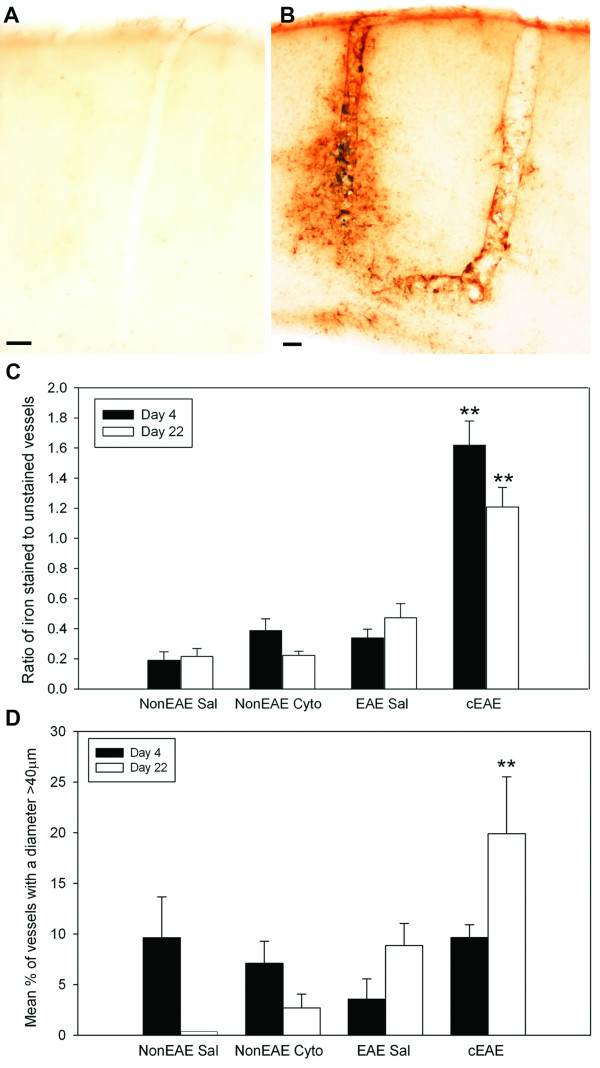
**Iron stained blood vessels are prevalent in cEAE**. **(A) **Control NonEAE Saline animals demonstrated little to no iron staining in the cortex. **(B) **cEAE animals revealed iron staining associated with vessels in the cortex. Staining was localized to the lining of vessels, the surrounding neuropil, and macrophages/microglia. Bars in A and B = 50 μm. **(C) **At both Day 4 and Day 22 (range 21-23) cEAE animals demonstrated a significantly higher ratio of iron stained vessels to unstained vessels as compared to NonEAE Saline animals. **(D) **At Day 22 (range 21-23) the mean percent of stained vessels in cEAE animals had a greater diameter than control NonEAE Saline animals (note, the bar slightly above 0, without an error bar, indicates analysis of tissue in this group of animals, but no labeled vessels were > 40 μm). Error bars = SEM. N = 6 for all groups, except cEAE Day 4 (N = 5). **p ≤ 0.01.

The spatial distribution of pathological iron deposits aligned with the distribution of the abnormal T2 hypointense areas (Figure 1). Iron deposits were observed within inflammatory cells in the subcortical white matter (Figure 1), lining blood vessels in the cortex (Figures 1, 2), dispersed in the neuropil, and in cells surrounding the vessels (Figure 2B) in both hemispheres. Iron labeled cells surrounding vessels were positive for Iba-1, a marker of microglia (discussed below). In addition, extravasation of RBCs was also occasionally observed (not shown).

### Iron labeled blood vessels

**Figure 3:**
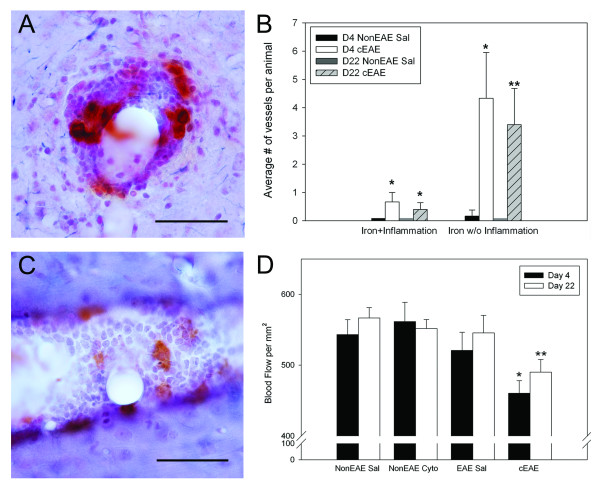
**Vascular changes in cEAE mice**. **(A) **A vessel with both inflammatory cells and iron staining. Bar = 50 μm. **(B) **The average number of vessels stained positive for iron with or without inflammation was significantly higher in cEAE mice than NonEAE Saline controls (note, bars slightly above 0, without error bars, indicate analyses of tissues in these groups of animals, but no iron stained vessels were observed). Also, the iron laden vessels without inflammation were significantly more abundant than iron laden vessels associated with inflammation. **(C) **A vessel from a cEAE mouse is congested with inflammatory cells as revealed by hematoxylin staining. Several iron labeled cells are also present in the vessel lumen. Bar = 50 μm. **(D) **Blood flow was decreased in both Day 4 and Day 22 (range 21-23) cEAE mice compared to control NonEAE Saline mice. Error bars = SEM. N = 6 animals per group, except Day 4 cEAE (N = 5). *p ≤ 0.05, **p ≤ 0.01.

Mice with cEAE had more iron stained vessels than control groups at both 4 and 21-23 days after the intracerebral injection (Figure 2C). Furthermore, the diameter of the iron stained vessels was increased in cEAE mice compared to the NonEAE saline control animals at Day 21-23, but not at Day 4 (Figure 2D). The majority of iron labeled vessels were not associated with inflammatory cells (Figure 3B), although inflammatory cells around vessels could be associated with (Figure 3A) or independent of (not shown) iron labeling of vessels in cEAE mice.

### Cerebral blood flow

Compared to NonEAE saline controls, mice with cEAE had significantly reduced cerebral blood flow (Figure 3D), which could be due to vessel congestion caused by inflammatory cells (Figure 3C). Increases in vessel diameter (Figure 2D) could also reflect consequences of vessel congestion.

### Perivascular microglial reactions

**Figure 4:**
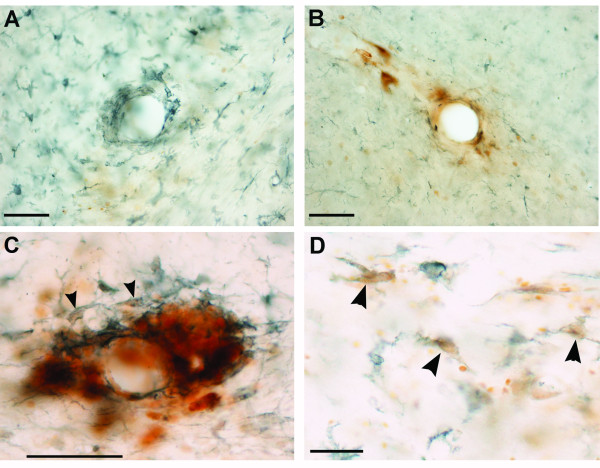
**Microglial Iba-1 and iron histochemical double staining in cEAE mice**. **(A) **Reactive microglia Iba-1 staining could be present around vessels without associated iron deposits, or conversely, **(B) **iron staining could be present around vessels without associated microgliosis. **(C) **An iron labeled vessel with associated microgliosis and double labeled cells revealing iron deposits within reactive microglia (arrowheads). **(D) **Reactive microglial cells containing iron deposits (arrowheads). Bars = 50 μm.

Iba-1 immunohistochemistry labeled reactive microglia with minimal background staining. Microgliotic reactions were present in both gray and white matter regions in both hemispheres and were often associated with vessels, but were not limited to these structures. Reactive microglia were more pronounced in mice with cEAE than in NonEAE saline control animals (not shown). Microgliotic reactions around vessels could be associated with iron deposits (Figure 4C), which were often localized within reactive microglia (Figure 4D), or microgliosis could be independent of iron deposits (Figure 4A). Alternatively, iron deposits around vessels could be independent of microgliosis (Figure 4B).

### Perivascular astrocyte reactions

**Figure 5:**
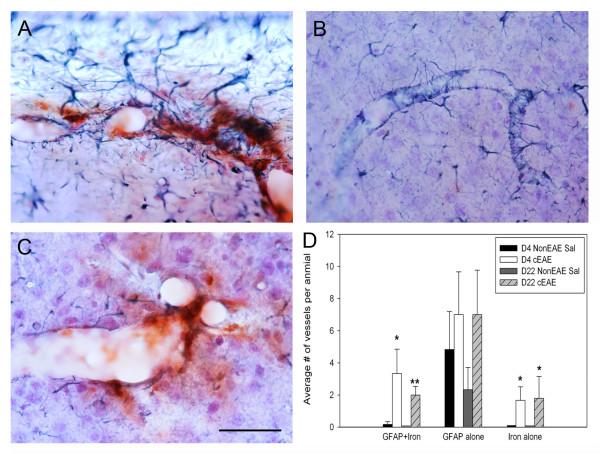
**GFAP, iron histochemical and hematoxylin staining**. **(A) **In cEAE mice, iron staining around vessels could be associated with astrocyte gliosis. On rare occasions, astrocytes were colabeled with iron (not shown). **(B) **In cEAE mice, GFAP staining around blood vessels could be observed independent of iron deposits, and conversely, **(C) **iron staining around vessels could be observed independent of astrocyte gliosis (note, the diminished density of dark blue astrocyte processes around the iron labeled vessel compared with A). Bar = 50 μm for A-C. **(D) **For all types of stained vessels (GFAP + iron, GFAP alone, iron alone), there was more staining in the cEAE mice compared to the NonEAE Saline controls, although the GFAP alone staining did not reach significance (note, bars slightly above 0 without error bars indicate analyses of tissues in these groups of animals, but values were 0). Error bars = SEM. N = 6 animals per group, except Day 4 cEAE (N = 5). *p ≤ 0.05, **p ≤ 0.01.

GFAP immunohistochemical staining revealed astrocyte gliosis in both gray and white matter structures in both hemispheres, and these reactions were often associated with vessels. Astrogliosis around vessels could be associated with (Figure 5A) or independent of (Figure 5B) iron deposition, in fact, the later was more common (Figure 5D). However, when gliosis was associated with iron deposits, it was more abundant in mice with cEAE compared to NonEAE saline controls at 4 and 21-23 days. In addition, iron deposits around vessels could occur independent of astrocyte gliosis (Figure 5C).

## Discussion

Perivenular pathology, such as the breakdown of the BBB, perivascular infiltration of inflammatory cells into the CNS, and perivenous demyelination are consistently identified in MS [12,14-16,24]. Despite these observations, a broader understanding of the contribution of vascular changes to the overall disease course has been lacking. Some studies suggest that MS patients suffer from extracranial vascular stenosis that affects blood flow patterns and CNS vessel drainage [3,4,8,11]; however, recent studies have raised doubts about these findings [25,26]. A hypothesis that linked observations on venous reflux to the generation of perivenular iron deposits [4,8,9,27], also proposed that the source of perivenular iron deposits is extravasated RBCs that occur as a consequence to alterations in blood flow patterns resulting in micro bleeds. The perivenular iron deposits were hypothesized to stimulate the infiltration of inflammatory cells into the CNS that induce subsequent pathological changes that are associated with MS, e.g., demyelination [4,8-10].

In order to test whether iron deposits initiate the inflammatory events that develop in MS, it is necessary to study the early stages of disease pathogenesis. However, these stages are difficult to study in humans due to the delay between the onset of symptoms and diagnosis, and the inability to obtain tissue specimens. EAE has been a useful tool for analyzing immune responses, testing potential therapeutics, and studying the development of pathology relevant to MS [17]. However, most EAE models develop spinal cord and brain stem pathology while cerebral pathology is limited and/or inconsistent. Since the hypothesis attributing inflammation as a consequence of iron deposition is largely based on analysis of cerebral tissue [3-10,16,18,27], an EAE model that consistently develops cerebral pathology is necessary to evaluate this proposition. Thus, we developed the cEAE model following the general approach used by Kerschensteiner and colleagues, i.e., an injection of cytokines to direct the inflammatory response to a specific CNS region [22]. This model reproduced many vessel-associated pathological features that are seen in MS including the accumulation of iron deposits around vessels. In addition to lining vessels, iron deposits were found in the surrounding neuropil, as extravasated RBCs, and within reactive microglia and macrophages. Although inflammatory cell infiltrates could be associated with perivascular iron deposits, inflammatory cells were also observed without associated iron deposits. This finding suggests that the development of inflammatory cell infiltrates is not dependent on iron deposition, which is in contrast to the hypothesis stating that iron deposits induce the inflammatory cell response that goes on to cause pathology such as demyelination.

Studies have shown the presence of iron accumulation around blood vessels in MS subjects using iron histochemistry [5,16,18] or MRI-SWI [6,7], but there has not been a direct correlation on the same specimens. In addition, T2 hypointense areas in gray matter structures from MS patients have been inferred to be iron deposits [28], but it has been difficult to establish a definitive link between histochemical findings and MRI observations due to the lack of brain tissue, for histochemical staining, from subjects who had an MRI just prior to death. In the present study, MRI T2-hypointense areas were compared with histochemical staining in the same mouse brain. Colocalizations of T2 hypointense areas and iron deposits were observed around blood vessels and in subcortical white matter. Other studies have utilized macrophages that were preloaded with exogenous iron to detect their accumulation within the CNS by MRI T2 hypointense areas in EAE subjects [29], but here we show the accumulation of endogenous iron deposits are colocalized to T2 hypointense areas.

In cEAE, pathological changes occurred rapidly after the onset of disease and were not routinely present in deep gray matter structures. Thus, these findings might not be applicable for addressing the accumulation of iron in deep gray matter structures in MS subjects that appears to advance as a result of long-standing disease. For example, iron deposition in deep gray matter structures is more pronounced in secondary progressive MS compared to relapsing remitting MS [28,30].

The presence of iron deposits around cerebral blood vessels was observed predominately in mice with cEAE, not in control groups. This indicates encephalitogenic activation of inflammatory cells and their further enticement by cytokines are required to initiate the vessel pathology seen in the cerebrum in our model. Iron deposits around cerebral vessels were also noted in another demyelinating disease, the twitcher mouse model of globoid cell leukodystrophy [31]. This disease is due to a genetic mutation in the glactosylceramidase gene, and thus, the accumulation of vessel associated iron deposits is secondary to the underlying genetic defect that results in demyelination. However, in addition to extensive demyelination, there are profound macrophage [32] and astrocyte gliotic [33] responses, and there is an enhanced expression of proinflammatory cytokines, e.g., TNF-α and IL-6 [34]. Thus, twitcher mice, cEAE mice and MS subjects all display iron deposits around vessels in conjuncture with the presence of proinflammatory cytokines [34-36] and activated inflammatory cells in the CNS [12,16]. Therefore, it appears that inflammatory stimuli can induce iron deposition around vessels independent of vessel stenosis. This would allow for an alternate mechanism for the development of iron deposition as put forward in the hypothesis proposing that vessel stenosis is the cause of perivenular iron deposits in brains of MS subjects.

Both astrogliosis and microgliosis were present in the brains of cEAE mice, and iron deposits were colocalized within microglia. However, iron deposits were not required for the development of astrogliosis and microgliosis around vessels since these reactions could be observed in the absence of perivascular iron deposits. On occasion, perivascular iron deposits appeared to induce a localized microgliotic reaction that was greater than that observed in the neighboring tissue (Figure 4C). It appears that these localized reactions were in response to extracellular iron deposits, such as those resulting from RBCs extravasation, but we cannot eliminate other types of iron deposits, e.g., those within endothelial or microglial cells. Other studies have shown that microglia cultured in the presence of excess iron have enhanced effector functions, increased NF-κB activity, and increased release of pro-inflammatory cytokines [37-40]. Since iron is taken up by perivascular microglia/macrophages in MS and EAE [5,12,18,20], it is possible that iron deposits could amplify pathogenesis via these cells. Iron deposits could also promote disease activity by acting as a catalyst in the production of reactive oxygen species, which have been implicated in disease progression of MS and EAE [41].

Disturbances to cerebral blood flow were observed in mice with cEAE. Altered blood flow has also been observed in MS by MRI [1,2] and in EAE [42]. In cEAE, we have observed vessel congestion, e.g., inflammatory cells filling vessels (Figure 3C), and suggest that this accounts, at least in part, for the reduced cerebral blood flow. Vessel congestion has also been noted in MS [12,14]. Vessel swelling is also observed in cEAE at 21-23 days, which may occur as a compensatory response to the vessel congestion.

## Conclusion

In summary, cEAE mice have iron deposits around vessels that can be seen by MRI and histochemistry. With time, these iron stained vessels developed a greater average diameter than control vessels, perhaps in response to inflammatory cell congestion leading to the observed decreased cerebral blood flow. Mice with cEAE also demonstrate increased astrogliosis and microgliosis, with reactive microglia co-labeling with iron deposits. In contrast to the hypothesis proposing iron deposition accounts for cellular inflammation, our data supports a pathogenic mechanism whereby vascular inflammation leads to alterations in CNS blood flow and iron deposition, not the other way around.

## Competing interests

Dr. Berman serves as a consultant for MAP Pharmaceuticals, has grants funded or pending with NIH and Merck, and has received payment from the National Headache Foundation. Dr. LeVine has received input, an honorarium, past and pending grant support and payment for travel from ApoPharma, Inc. Dr. LeVine has also received 1) grant support from the NIH and Heartland Border Walk for MS, 2) royalties from the Taylor and Francis group, and 3) payment for reviewing grants for the University of California Office of the President, Tobacco-Related Disease Research Program. Dr. Lynch has a grant funded or pending with ApoPharma, Inc., and the National Multiple Sclerosis Society and has received financial support from Fondazione Hilarescare to travel to a meeting on chronic cerebrospinal venous insufficiency.

## Authors' contributions

RW helped design the studies and carried out the EAE induction and cerebral injections, the laser Doppler, vessel diameter measurements, iron histochemistry, and contributed significantly to writing the manuscript. AMR carried out the GFAP and Iba-1 staining and analysis. WTW, ICY, and PL carried out the MRI studies and analysis. NEJB and SGL discussed the project and made critical revisions to the manuscript. SML designed the studies, helped analyze the data, and played a substantial role in writing the manuscript. All authors read and approved the manuscript.
